# Genome-wide identification and characterization of long non-coding RNAs during postnatal development of rabbit adipose tissue

**DOI:** 10.1186/s12944-018-0915-1

**Published:** 2018-11-28

**Authors:** Guo-Ze Wang, Kun Du, Shen-Qiang Hu, Shi-Yi Chen, Xian-Bo Jia, Ming-Cheng Cai, Yu Shi, Jie Wang, Song-Jia Lai

**Affiliations:** 1 Farm Animal Genetic Resources Exploration and Innovation Key Laboratory of Sichuan Province, Sichuan Agricultural University, Chengdu, 611130 China; 2 College of Pharmacy and Biological Engineering, Chengdu University, Chengdu, 610106 China

**Keywords:** Rabbit, Adipose tissue, Long non-coding RNA, RNA-Seq

## Abstract

**Background:**

The rabbit is widely used as an important experimental model for biomedical research, and shows low adipose tissue deposition during growth. Long non-coding RNAs (lncRNAs) are associated with adipose growth, but little is known about the function of lncRNAs in the rabbit adipose tissue.

**Methods:**

Deep RNA-sequencing and comprehensive bioinformatics analyses were used to characterize the lncRNAs of rabbit visceral adipose tissue (VAT) at 35, 85 and 120 days after birth. Differentially expressed (DE) lncRNAs were identified at the three growth stages by DESeq. The *cis* and *trans* prediction ways predicted the target genes of the DE lncRNAs. To explore the function of lncRNAs, Gene Ontology (GO) enrichment and Kyoto Encyclopedia of Genes and Genomes (KEGG) pathway analyses were performed on the candidate genes.

**Results:**

A total of 991,157,544 clean reads were generated after RNA-Seq of the three growth stages, of which, 30,353 and 107 differentially expressed (DE) lncRNAs were identified. Compared to the protein-coding transcripts, the rabbit lncRNAs shared some characteristics such as shorter length and fewer exons. *Cis* and *trans* target gene prediction revealed, 43 and 64 DE lncRNAs respectively, corresponding to 72 and 20 protein-coding genes. GO enrichment and KEGG pathway analyses revealed that the candidate DE lncRNA target genes were involved in oxidative phosphorylation, glyoxylate and dicarboxylate metabolism, and other adipose growth-related pathways. Six DE lncRNAs were randomly selected and validated by q-PCR.

**Conclusions:**

This study is the first to profile the potentially functional lncRNAs in the adipose tissue growth in rabbits, and contributes to our understanding of mammalian adipogenesis.

**Electronic supplementary material:**

The online version of this article (10.1186/s12944-018-0915-1) contains supplementary material, which is available to authorized users.

## Background

Obesity is becoming increasingly prevalent in both developed and developing countries, and the proportion of overweight and obese individuals is expected to reach 89 and 85% in men and women respectively,by 2030 [1, 2]. Since excessive weight gain is associated with diseases of the metabolic syndrome, including hyperglycemia, dyslipidemia, hypertension, atherosclerosis [3, 4], diabetes and cardiovascular disease, the global rise in obesity poses a considerable threat to human health [5, 6]. Obesity manifests as over-accumulation of fat in white adipose tissue (WAT), due to an increase in the size and number of adipocytes [7]. WAT is categorized into two types based on its distribution: the visceral adipose tissue (VAT) which is located within specific regions of the abdominal cavity, and subcutaneous adipose tissue (SAT), which is present below the skin [8]. The amount of fat in both VAT and SAT is closely related to the incidence of metabolic diseases [9], and excessive fat accumulation in VAT in particular is regarded as a high risk factor for metabolic disorders and cardiovascular diseases [10–12]. Therefore, studies on visceral adipocyte differentiation and its potential regulatory mechanisms have long been the core of obesity research. The development and function of VAT is age dependent, as one study showed that protein synthesis in bovine adipose tissue was more active in fetuses than in adults [13], as were the transcription factors PPARG, C/EBP and STAT which are known to promote the development of WAT [14].

Non-coding RNAs (ncRNAs) are functional RNAs that do not encode proteins, but play important roles in various cellular processes [15–17]. Based on their functions and lengths, ncRNAs are classified into different categories including microRNAs (miRNAs), transfer RNAs (tRNAs), small interfering RNAs (siRNAs), ribosomal RNAs (rRNAs), and the long non-coding RNAs (lncRNAs) which are > 200 nucleotides long [18–20]. With the development of high-throughput technologies like RNA-seq, large-scale expression analysis has accelerated the discovery and characterization of lncRNAs [21, 22]. The latter plays critical roles in many biological processes such as epigenetic modification [23, 24], gene transcription [23, 25], cell differentiation [24, 26], growth and development, as well as in some obesity-related diseases [27].

Rabbits are an economically important livestock animal, and are raised for meat and fur production, as well as experimental models for biomedical research. Rabbit meat is protein rich, and low in cholesterol, and fat. Since the adipose tissue in rabbits has a lower deposition rate during growth, the rabbit is an ideal model to study adipose regulation [28–32]. Several lncRNAs involved in adipogenesis in mammals like cattle [33], pigs [34] and chicken [35], have been identified but the adipose tissue growth-related lncRNAs in rabbits have not been profiled so far.

We used the RNA-seq based approach to determine lncRNAs expression levels in rabbit VAT at 35, 85 and 120 days after birth, and identified 30,353 lncRNAs of which 107 were differentially expressed indicating their potential in rabbit adipogenesis. Our findings provide further insights into the regulatory function of lncRNAs in rabbits and for annotating the rabbit genome, as well as contribute to better understanding of adipogenesis.

## Materials and methods

### Animal and sample collection

Tianfu Black rabbits (native species in Sichuan province of China) aged 35, 85 and 120 days were used in this study. Given the plasticity and maturation of rabbit VAT [36], three biological replicates of peri-renal fat were collected for 35 days (YR) and 120 days (TR), and two for 85 days (MR). The samples were snap frozen in liquid nitrogen, and stored at − 80 °C until RNA extraction.

### Total RNA extraction

Total RNA was isolated using Trizol Reagent (Life Technologies, Carlsbad, CA, USA). The purity and integrity of the RNA were determined using Nanodrop (Thermo Fisher Scientific, Waltham, MA, USA) and Agilent Bioanalyzer 2100 system (Agilent Technologies, CA, USA) respectively, and RNA concentration was measured using a Qubit® RNA Assay Kit and Qubit® 2.0 Fluorometer (Life Technologies, Carlsbad, CA, USA). Integrity of RNA was assessed using. Only samples that had RNA Integrity Number scores > 8 were used for sequencing.

### Library construction and sequencing

The libraries construction and sequencing were performed by Mega Genomics Co.,Ltd., (Beijing, China). Briefly, 1 μg RNA was taken per sample and rRNA was removed using a NR603-VAHTS Total RNA-seq (HMR) Library Prep Kit (Vazyme Biotech Co.,Ltd., Nanjing, China). First-strand cDNA was synthesized using random hexamer primers, followed by the second-strand synthesis using DNA polymerase I and RNase H. The resulting double-stranded DNA was purified by AMPure XP beads, and a poly A tail was ligated to the sequencing joint. The correct-sized fragments were purified by AMPure XP beads. The USER enzyme was used to degrade the cDNA strands containing U instead of T, and the first-strand cDNA was sequenced, thereby preserving the direction of the RNA. Finally, PCR amplification was conducted and the products were purified (AMPure XP beads) for constructing the cDNA libraries.

The quality of the latter was assessed using Agilent BioAnalyzer 2100 system and qPCR. The libraries were sequenced on an Illumina HiSeq X Ten platform and 150-bp long paired-end reads were generated.

### Transcriptome assembly and lncRNAs detection

To ensure the accuracy of information analysis, the adapter and low-quality reads were removed from the raw reads, and only the high quality clean reads were used for subsequent analysis. They were aligned to the rabbit reference genome (GCF_000003625.3_OryCun2.0_genomic.fa), along with annotated genes (GCF_000003625.3_OryCun2.0_genomic.gff) using histat2 (2.0.5) software with the parameters ‘-dta-rf-p 1-x-1-1-S File for SAM output (default:stdout)’ [37]. The StringTie program [38, 39] was used to splice the Mapped Reads. LncRNA identification consisted of two steps: basic screening and potential coding ability screening. The transcripts longer than 200 bp, containing two or more exons, and fragments per kilobase of transcript per million fragments mapped (FPKM) [40] ≥ 0.1 were first selected by basic screening. Subsequently, CPC [41], CNCI [42], CPAT [43], and Pfam protein structure domain analysis [44] were used to screen for potential coding transcripts. Stringtie (1.3.3) was used to quantify transcripts and normalize the expression values (FPKM).

### Screening of differentially expressed lncRNAs

Differentially expressed (DE) lncRNAs between any two libraries were identified by DESeq (1.26.0), with padjust < 0.01 and an absolute value of the |log2(fold change)| ≥ 2.0 or ≤ 1/2.0 as the threshold. All DE lncRNAs in eight libraries were clustered using the Heatmaps in R software package.

### Target gene prediction and functional enrichment analysis

Most of the lncRNAs deposited in current databases have not yet been functionally annotated. Therefore, prediction of their functions is based on the functional annotations of their related *cis* and *trans* mRNAs. Coding genes located at the distance of 100-kb were considered potentially *cis*-regulated target genes, while LncTar [45] was used to predict the potentially *trans*-regulated target genes of the DE lncRNAs. GO enrichment and KEGG pathway analyses of the candidate DE lncRNA target genes were then performed using GOseq [46] and R package, respectively. Significance was calculated using the Expression Analysis method, and *P* value < 0.05 was considered significant.

### Validation of DE lncRNAs by q-PCR

Primers for the lncRNAs and internal controls (Additional file 1) were designed using Primer-BLAST (https://www.ncbi.nlm.nih.gov/tools/primer-blast/). Total RNA was converted to cDNA using a PrimeScript™ RT Reagent Kit containing gDNA Eraser (TAKARA, Dalian, China), and oligo (dT) and random hexamer primers. The q-PCR was performed using SYBR Premix Ex Taq™ II (TAKARA) according to the manufacturer’s instructions. The reaction mix consisted of 5 μl SYBR Premix Ex Taq™ II, 1 μl template cDNA, 0.4 μl of 10 μM forward and reverse primers, and 3.2 μl dH2O to a final volume of 10 μl. The reactions were performed on a Rotor gene 6000 PCR System (QIAGEN, Hiden, Germany) as follows: 95 °C for 10s, followed by 40 cycles of 95 °C for 5 s, and 20s at the Tm (Additional file 1). Melting curve analysis was performed from 65 °C to 95 °C with increments of 1.5 °C. The expression levels of lncRNAs were normalized to HPRT1 and GAPDH. Relative gene expression levels were calculated using the 2^-ΔΔCt^ method [47], and data were expressed as mean ± standard error of the mean (SEM).

### Statistical analysis

Statistical analysis was performed using the SPSS Statistics 20.0 (SPSS Inc., Chicago,

IL, USA), and *P* < 0.05 was considered statistically significant.

## Results

### Overview of RNA-Seq

To identify lncRNAs expressed during the growth of rabbit adipose tissue, we constructed 8 cDNA libraries (YR-1,YR-2,YR-3, MR-1,MR-2,TR-1,TR-2 and TR-3) from the peri-renal adipose tissues of 35 (YR), 85 (MR), and 120 (TR) day-old rabbits. The libraries were sequenced using the Illumina HiSeq X Ten platform, and 1,016,066,842 raw reads were generated. After filtering adaptor sequences and low-quality reads, we finally obtained 991,157,544 clean reads. The percentage of clean reads and GC content of libraries ranged from 97.29–98.12% and 49.5–51.65%. After mapping the clean reads to the rabbit reference genome, approximately 89.53% of the clean reads were selected for further analysis (Additional file 2).

### Characterization of lncRNAs in rabbit adipose tissue

**Fig. 1:**
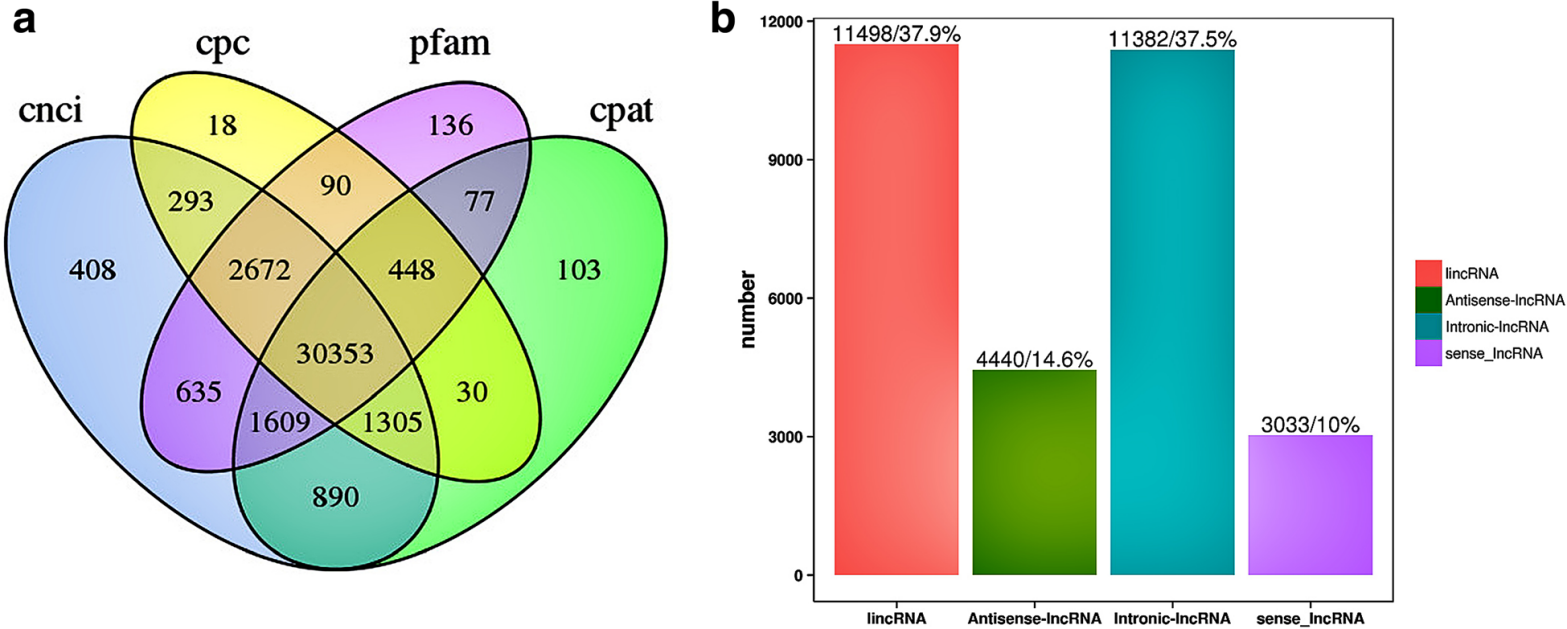
Identification of rabbit lncRNAs. **a** The Venn diagram of lncRNA transcripts from four tools CNCI (Coding-Non-Coding Index), CPC (Coding Potential Calculator), PFAM (the Protein Families Database), and CPAT (Coding Potential Assessment Tool). The lncRNAs identified by all four analytical tools were used in subsequent analyses. **b** The number of the four lncRNA types

**Fig. 2:**
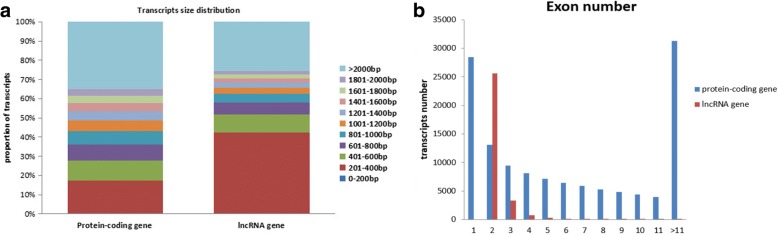
Comparison between rabbit lncRNAs and protein-coding genes. **a** Transcript size distribution of rabbit lncRNAs and protein-coding genes. **b** Exon numbers of rabbit lncRNAs and protein-coding genes

**Fig. 3:**
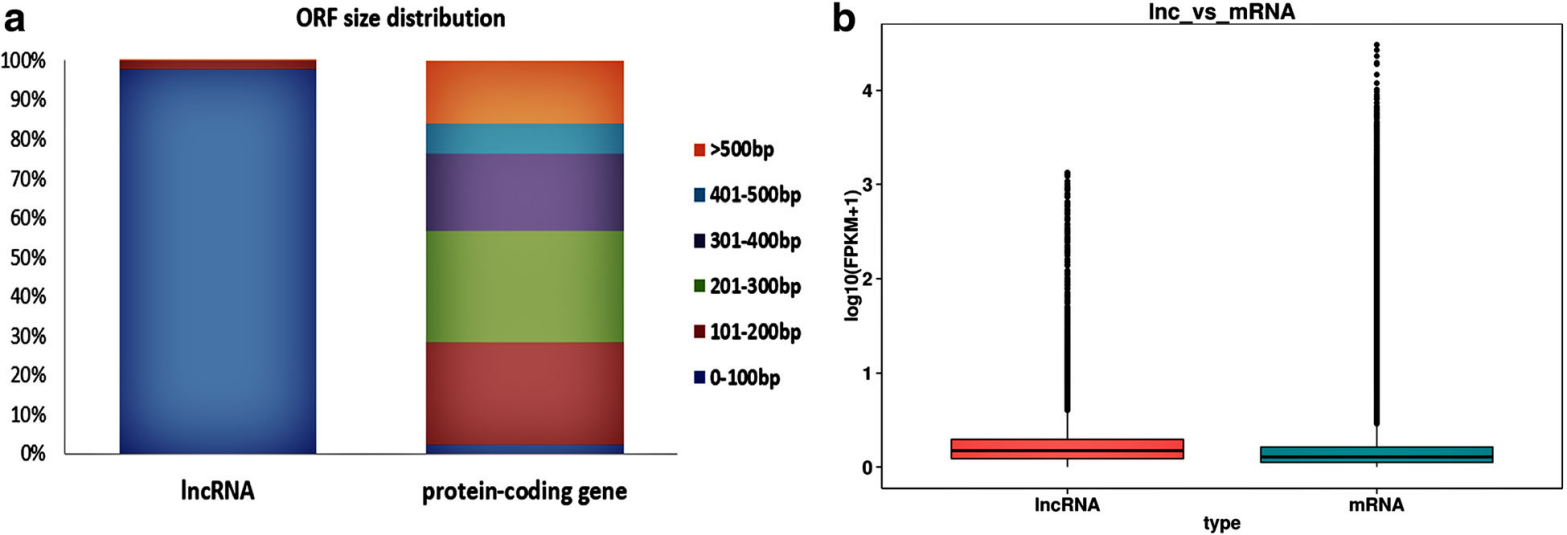
The distribution of open reading frame(ORF) size and expression level of the lncRNAs and protein-coding genes. **a** Distribution of ORF sizes in the lncRNAs and protein-coding genes. **b** Expression level analysis in the lncRNAs and protein-coding genes

The RNA-seq produced 30,353 lncRNAs (Fig. 1a, Additional file 3) and 119,502 protein-coding transcripts. The lncRNA transcripts included 11,498 lincRNAs(37.9%), 4440 anti-sense lncRNAs (14.6%), 11,382 intronic lncRNAs (37.5%) and 3033 sense lncRNAs (10%) (Fig. 1b).The average length of the lncRNAs was considerably shorter compared to that of the protein-coding genes (Fig. 2a). Furthermore, the protein-coding genes with average 7.8 exon numbers were more than the lncRNAs with average 2.3 exons (Fig. 2b), and the open reading frame (ORF) size in the protein-coding genes was longer than the ORFs of lncRNAs(most of lncRNAs were within 100 bp) (Fig. 3a). In addition, lncRNAs exhibited a lower level of expression than the protein-coding genes (Fig. 3b).

### Identification of DE lncRNAs

**Fig. 4:**
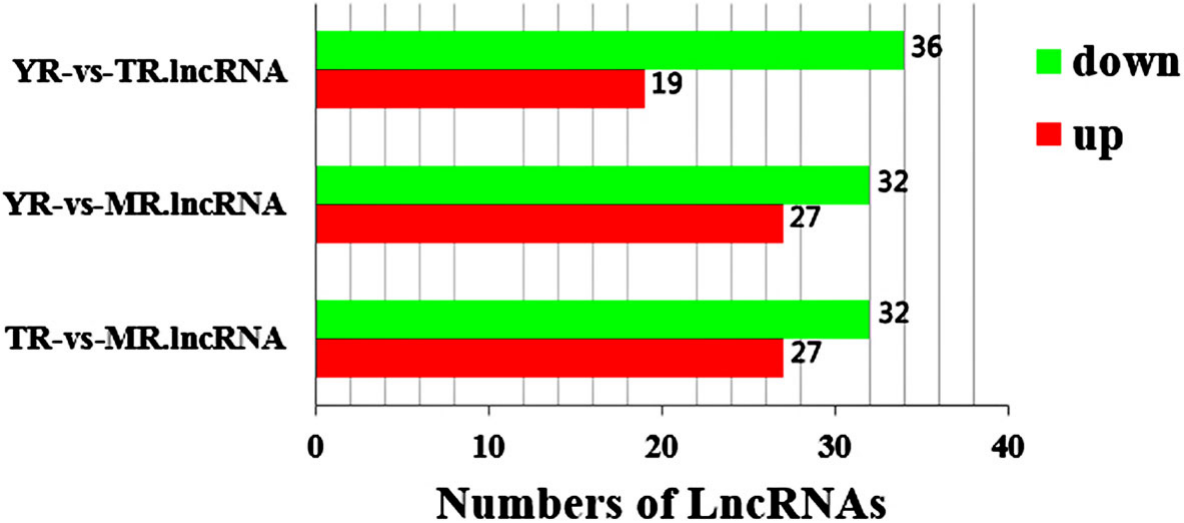
Number of up-regulated and down-regulated lncRNAs in the rabbit peri-renal adipose at three growth periods

**Fig. 5:**
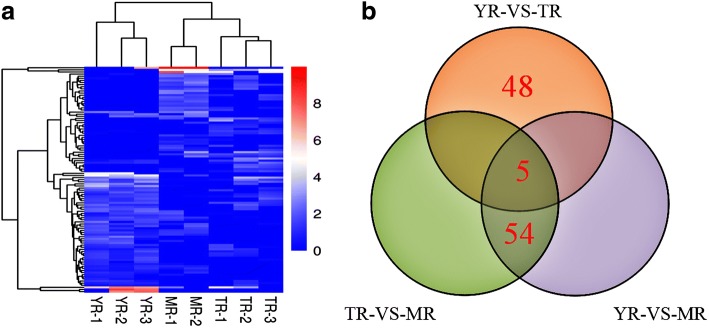
Analyses of DE lncRNAs in the RNA-Seq libraries. **a** Hierarchical clustering analysis of lncRNA expression profiles from 8 libraries with 107 DE lncRNAs.Data are expressed as FPKM.Red - relatively high expression, Green - relatively low expression. **b** Venn diagram showing the DE lncRNAs at the three growth periods

The expression levels of the lncRNAs were calculated by FPKM using DESeq. A total of 107 DE lncRNAs were identified (*p* < 0.05) during the growth of adipose tissue (Fig. 4), of which 42.7% were up-regulated and 57.3% were down-regulated. The DE lncRNAs with similar expression levels across the different libraries were screened and clustered using the systematic cluster analysis (Fig. 5a). Pairwise comparison of the YR, MR and TR lncRNA data showed 59, 59 and 53 DE lncRNAs between the respective growth stages. A Venn diagram constructed using these DE lncRNAs showed that 54 DE lncRNAs overlapped between YR vs MR and TR vs MR comparisons, while 5 DE lncRNAs (MSTRG.131023.1, MSTRG.258713.8, MSTRG.262225.9, MSTRG.80672.4, MSTRG.93331.2) were common to all three growth stages (Fig. 5b).

### Enrichment analysis of the target genes of DE lncRNAs

**Fig. 6:**
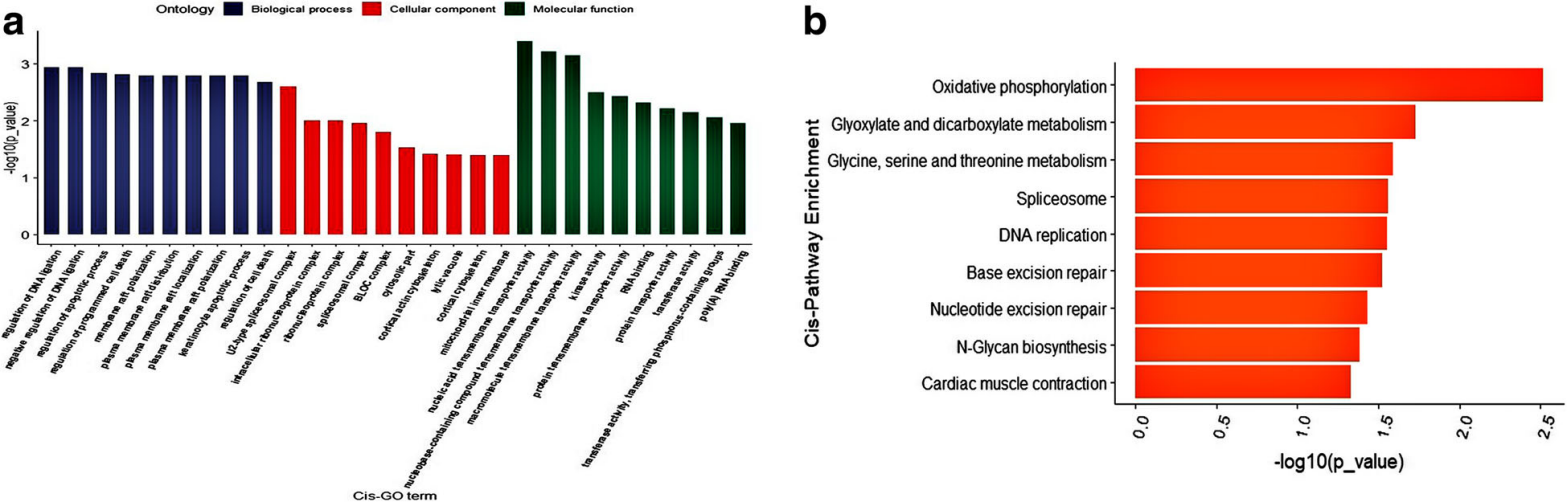
GO enrichment analysis and KEGG pathway enrichment analysis of the *cis*-regulated target genes. **a** The top 10 significant BP, MF and CC terms in GO enrichment analysis at *p*-value < 0.05. **b** The KEGG enrichment analysis, with the vertical axis showing the significantly enriched pathways with *p*-value < 0.05

The potential *cis*-regulated target genes of lncRNAs were first predicted and 43 out of the 107 DE lncRNAs corresponded to 72 protein-coding genes. Gene ontology (GO) analysis [48] of the *cis* lncRNA targets showed significant enrichment of 192 GO terms (*P* < 0.05), of which U2-type spliceosomal complex (GO:0005684), nucleic acid transmembrane transporter activity (GO:0051032) and regulation of DNA ligation (GO:0051105) were the top listed GO terms involved in cellular component (CC), molecular function (MF) and biological process (BP) respectively (Fig. 6a). In addition, several terms associated with adipose accumulation like cell morphogenesis (GO:0000902), macromolecule metabolic process (GO:0043170) and lytic vacuole (GO:0000323) were also highly enriched. Furthermore, 20 Kyoto Encyclopedia of Genes and Genomes (KEGG) pathways were enriched, of which several were related to adipocyte growth such as oxidative phosphorylation (KO00190), glyoxylate and dicarboxylate metabolism (KO00630), and calcium signaling pathway (KO4323) (Fig. 6b). Interestingly, we also found target genes including *GCSH*, *NDUFS5*, *HNRNPA3*, *GSN*, *CYSLTR2,* and *TFIP11*, which were annotated with adipose growth-related GO terms and pathways.

**Fig. 7:**
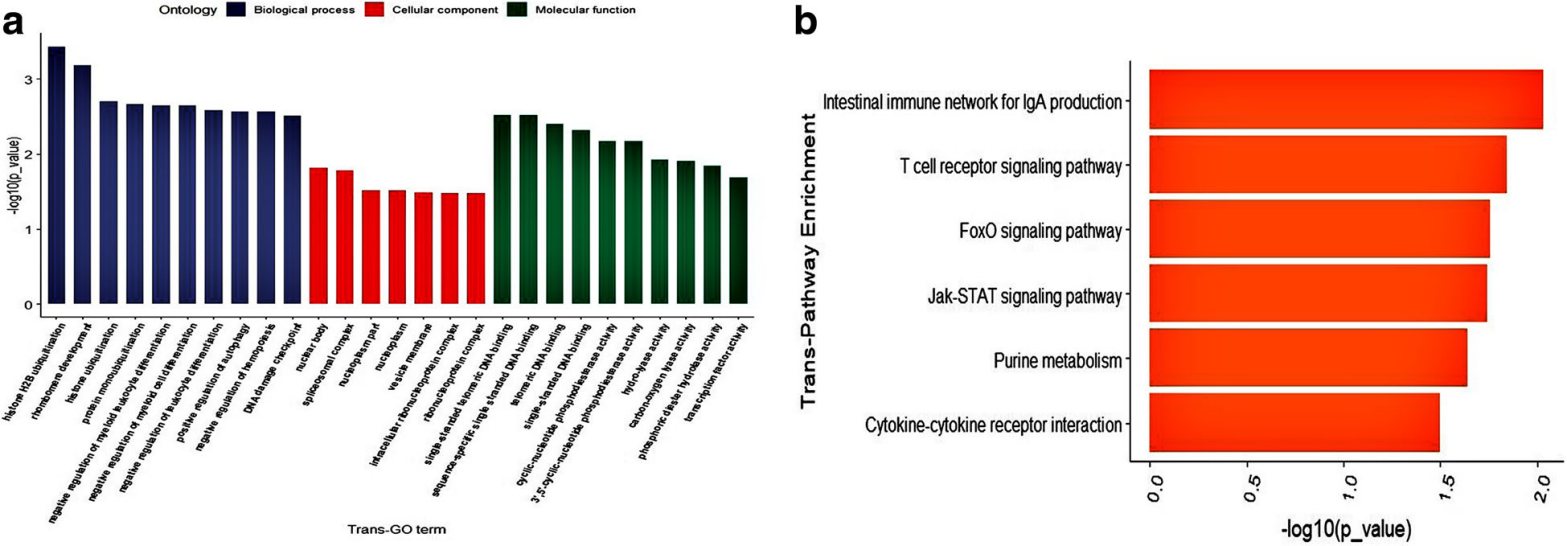
GO enrichment analysis and KEGG pathway enrichment analysis of the *trans*-regulated target genes. **a** The top 10 significant BP, MF and CC terms of GO enrichment analysis at *p*-value < 0.05. **b** The KEGG enrichment analysis, with the vertical axis showing the significantly enriched pathways with *p*-value < 0.05

Prediction of the *trans*-regulated target genes showed that, 64 DE lncRNAs corresponded to 20 protein-coding genes, with significant enrichment of 124 GO terms (*P* < 0.05), including carbon-oxygen lyase activity (GO:0016835), regulation of catabolic process (GO:0009894) and cell cycle process (GO:0022402) (Fig. 7a). KEGG analysis revealed 5 enriched pathways (Fig. 7b) including the Jak-STAT signaling (KO04630) and Purine metabolism pathways (KO00230). Taken together, the majority of target genes of the DE lncRNAs in different growth stages were related to adipose development and growth.

### Validation of DE lncRNAs

**Fig. 8:**
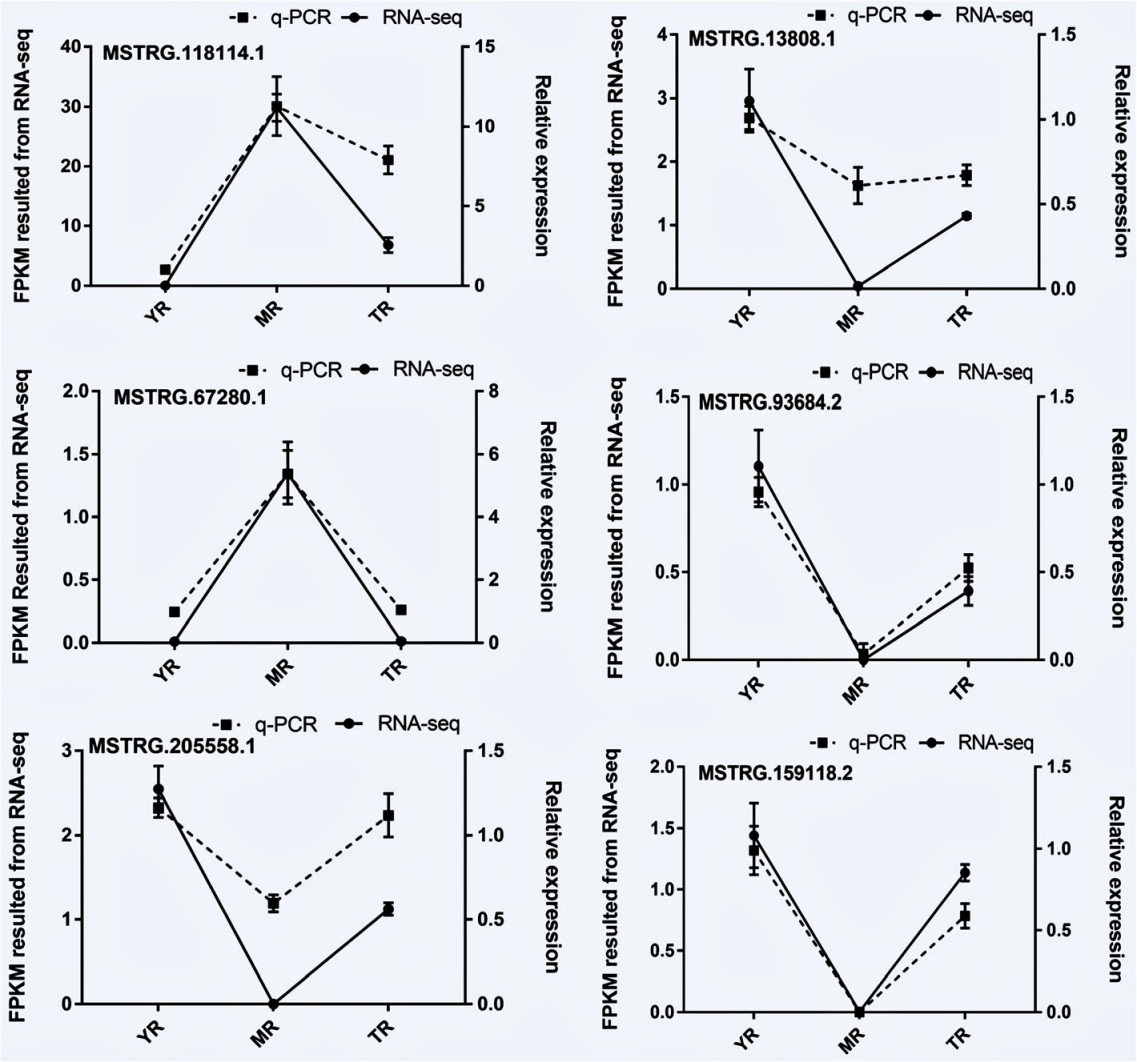
Validation of six randomly selected DE lncRNAs by q-PCR

To validate the RNA-Seq results, we randomly selected six DE lncRNAs and examined their expression patterns at the three growth stages by q-PCR. The results showed that all six lncRNAs (MSTRG.118114.1, MSTRG.93684.2, MSTRG.13808.1, MSTRG.20558.1, MSTRG159118.2, MSTRG.67280.1) were differentially expressed at different stages. In addition, the six lncRNAs exhibited a similar trend between the results of RNA-seq and q-PCR (Fig. 8). Therefore, the FPKM obtained from RNA-seq can be reliably used to determine lncRNAs expression.

## Discussion

LncRNAs play important roles in genomic imprinting, cellular trafficking and organization, as well as regulation of gene expression and dosage compensation [49]. However, in contrast to mice [50, 51] and humans [52], limited information is available regarding rabbit lncRNAs. We identified 30,353 lncRNAs in the rabbit peri-renal adipose tissues, which is more than the number reported for chicken [53, 54] and pigs [34]. This may be the result of the differences between the species. To the best of our knowledge, this is the first report to systematically identify lncRNAs at the three growth stages of rabbit VAT: 35, 85, and 120 days after birth using RNA-Seq. Six of these DE lncRNAs were validated using q-PCR, and showed similar trends as the RNA-Seq results, indicating that our approach was reliable and accurately reflected the differential lncRNA expression during adipose growth in rabbit.

Compared to previous studies on other species, the rabbit lncRNAs were shorter, and had fewer exons, shorter ORFs and lower expression levels compared to the protein-coding genes [55–57]. These characteristics of lncRNAs are common across mammals, and are likely significant to their regulatory function, in addition to acting as a template for identifying the lncRNAs of different mammalian species.

In this study, we detected a total of 107 DE lncRNAs in the pairwise comparisons of different growth stages of rabbit VAT. The numbers of down-regulated lncRNAs was higher than that of the up-regulated lncRNAs, indicating that most lncRNAs were involved in the early adipose growth. In addition, 54 lncRNAs were differentially expressed in both the YR vs MR and TR vs MR comparisons, although their expression levels were different. This indicates that the DE lncRNAs likely play different role in the different stages of VAT growth. Taken together, the DE lncRNAs identified in our study are important regulators of rabbit adipose growth.

Our and others’ studies were not able to infer the function of lncRNAs from their sequences and structures [58, 59]. LncRNAs regulate the expression of nearby or distal genes, respectively known as *cis* or *trans* regulation [60]. We predicted the potential *cis* and *trans* regulated target genes of the 107 DE lncRNAs, and determined their potential biological relevance using GO and KEGG analysis.

The *cis*-regulated target genes showed significant enrichment in GO terms associated with lipid metabolism and functions of adipose tissue, such as mRNA metabolic process, cellular protein modification process, cell part morphogenesis, mast cell cytokine production, histone H2B ubiquitination, response to corticotropin-releasing hormone, mast cell activation involved in immune response, macromolecule metabolic process, growth and lytic vacuole organization. Pathway analysis showed that the *cis*-target genes of the DE lncRNAs were mainly involved in oxidative phosphorylation, glyoxylate and dicarboxylate metabolism, glycine, serine and threonine metabolism, and calcium signaling pathway. Calcium is a key intracellular signaling mediator regulating numerous cellular processes [61]. Activation of CaSR, an extracellular Ca^2+^ sensor, in the visceral white adipose progenitor cells increases proliferation and adipocyte differentiation [62]. Some of the *cis* target protein-coding genes are involved in VAT growth, for e.g. *GCSH*, *NDUFS5*, *HNRNPA3*, *GSN*, *CYSLTR2,* and *TFIP11.* These results indicate the possible roles of the DE lncRNAs in regulating adipogenesis. Recent studies have shown that lncRNAs such as PU.1 antisense lncRNA, ADINR, lncRNA U90926 and NEAT1 directly or indirectly regulate expression of PPARγ [63–66]. The PU.1 antisense lncRNA was discovered within the mouse PU.1 locus [67], and promoted adipogenesis by transcriptionally repressing the adjacent PU.1 mRNA [63].

Some lncRNAs can also regulate their target genes in the *trans* mode [68], and the *trans*-regulated target genes predicted in this study were enriched in MF terms like immune system, signal transduction, and nucleotide metabolism and BP terms including cell cycle process, coenzyme metabolic process, regulation of cell differentiation and regulation of multicellular organismal development. Some of the *trans* target protein-coding genes were involved in VAT growth such as *IL10, PDE6D, MAFB,* and *PCBD2*. Large scale genomic studies in recent years have furthered our understanding of lncRNAs as functional adipogenic regulators [69]. *IL-10*, an anti-inflammatory cytokine is known to contribute to childhood obesity [70, 71], and Liu et al found that *IL-10* and the downstream JAK-STAT pathway were down-regulated in obese children with hypertriglyceridemia and in HFD obese rats [72]. This, indicated a protective effect of *IL-10* on lipid metabolic disorders, especially hypertriglyceridemia. Therefore, the DE lncRNAs might *trans*-regulate adipogenic differentiation and lipid metabolism by targeting genes involved in the relevant signaling pathways. In conclusion, we identified lncRNAs that regulate adipose growth and apoptosis through cis or trans-acting mechanisms. In future studies, we will investigate the functions of some of these DE lncRNAs in order to elucidate the regulatory mechanisms and potential therapeutic targets for obesity.

## Conclusions

This is the first report of the lncRNA profile of rabbit VAT at different stages of growth, which identified 107 DE lncRNAs associated with adipogenetic pathways like oxidative phosphorylation, cell part morphogenesis and glyoxylate and dicarboxylate metabolism. These DE lncRNAs are thus involved in the development and metabolism of adipose tissue in rabbits.

## Additional files

Additional file 1: The Primer information of lncRNA for qPCR validation (XLSX 11 kb)

Additional file 2: Overview of RNA-seq. (XLSX 10 kb)

Additional file 3: List of 30353 annotated lncRNA loci. (XLSX 1322 kb)

## Abbreviations

CC: Cellular component
DE: Differentially expressed
FPKM: Fragments per kilobase of transcript per million fragments mapped
GO: Gene ontology
KEGG: Kyoto Encyclopedia of Genes and Genomes
lncRNAs: Long non-coding RNAs
MF: Molecular function; BP: biological process
ORF: The open reading frame
SAT: Subcutaneous adipose tissue
VAT: Visceral adipose tissue
WAT: White adipose tissue

## References

[1] You W, Henneberg M (2018). Relaxed natural selection contributes to global obesity increase more in males than in females due to more environmental modifications in female body mass. PLoS One.

[2] Rtveladze K, Marsh T, Barquera S, Sanchez Romero LM, Levy D, Melendez G, Webber L, Kilpi F, Mcpherson K, Brown M (2014). Obesity prevalence in Mexico: impact on health and economic burden. Public Health Nutr.

[3] Sharma BR, Kim DW, Rhyu DY (2017). Korean Chungtaejeon tea extract attenuates weight gain in C57BL/6J-Lep Ob/Ob mice and regulates adipogenesis and lipolysis in 3T3-L1 adipocytes. J Integr Med.

[4] Wang WJ, Teng Z (2017). Integration of traditional Chinese medicine and Western medicine in the era of precision medicine. Journal of Integrative Medicine.

[5] Wang YC, Mcpherson K, Marsh T, Gortmaker SL, Brown M (2011). Health and economic burden of the projected obesity trends in the USA and the UK. Lancet.

[6] Rutter H, Bes-Rastrollo M, Henauw SD, Lahti-Koski M, Lehtinen-Jacks S, Mullerova D, Rasmussen F, Rissanen A, Visscher TLS, Lissner L (2017). Balancing upstream and downstream measures to tackle the obesity epidemic: a position statement from the European Association for the Study of obesity. Obesity Facts.

[7] Rosen ED, Macdougald OA (2006). Adipocyte differentiation from the inside out. Nat Rev Mol Cell Biol.

[8] Seale P, Conroe HM, Estall J, Kajimura S, Frontini A, Ishibashi J, Cohen P, Cinti S, Spiegelman BM (2011). Prdm16 determines the thermogenic program of subcutaneous white adipose tissue in mice. J Clin Investig.

[9] Arner P (2005). Human fat cell lipolysis: biochemistry, regulation and clinical role. Best Pract Res Clin Endocrinol Metab.

[10] Fox CS, Massaro JM, Hoffmann U, Pou KM, Maurovichhorvat P, Liu CY, Vasan RS, Murabito JM, Meigs JB, Cupples LA (2007). Abdominal visceral and subcutaneous adipose tissue compartments association with metabolic risk factors in the Framingham heart study. Circulation.

[11] Pischon T, Boeing H, Hoffmann K, Bergmann M, Schulze MB, Overvad K, Yt VDS, Spencer E, Moons KG, Tjønneland A (2008). General and abdominal adiposity and risk of death in Europe. J Vasc Surg.

[12] Wang Y, Rimm EB, Stampfer MJ, Willett WC, Hu FB (2005). Comparison of abdominal adiposity and overall obesity in predicting risk of type 2 diabetes among men. Am J Clin Nutr.

[13] Zhou Y, Sun J, Li C, Wang Y, Li L, Cai H, Lan X, Lei C, Zhao X, Chen H (2014). Characterization of transcriptional complexity during adipose tissue development in bovines of different ages and sexes. PLoS One.

[14] White UA, Stephens JM (2010). Transcriptional factors that promote formation of white adipose tissue. Molecular & Cellular Endocrinology.

[15] Chen K, Rajewsky N (2007). The evolution of gene regulation by transcription factors and microRNAs. Nat Rev Genet.

[16] Nissen P, Hansen J, Ban N, Moore PB, Steitz TA (2000). The structural basis of ribosome activity in peptide bond synthesis. Science.

[17] Zhang Y, Huang H, Zhang D, Qiu J, Yang J, Wang K, Zhu L, Fan J, Yang J. A Review on Recent Computational Methods for Predicting Noncoding RNAs. Biomed. Res. Int. 2017;(2017-5-3) 2017, 2017:9139504. 10.1155/2017/9139504 PMC5434267 28553651

[18] Lindgreen S, Gardner PP, Krogh A (2007). MASTR: multiple alignment and structure prediction of non-coding RNAs using simulated annealing. Bioinformatics.

[19] Nawrocki EP, Kolbe DL, Eddy SR (2009). Infernal 1.0: inference of RNA alignments. Bioinformatics.

[20] Wang XJ, Reyes JL, Chua NH, Gaasterland T (2004). Prediction and identification of Arabidopsis thaliana microRNAs and their mRNA targets. Genome Biol.

[21] Weikard R, Hadlich F, Kuehn C (2013). Identification of novel transcripts and noncoding RNAs in bovine skin by deep next generation sequencing. BMC Genomics.

[22] Cabili MN, Trapnell C, Goff L, Koziol M, Tazonvega B, Regev A, Rinn JL (2011). Integrative annotation of human large intergenic noncoding RNAs reveals global properties and specific subclasses. Genes Dev.

[23] Cao J: The functional role of long non-coding RNAs and epigenetics. Biological Procedures Online,16,1(2014-09-15) 2014, 16:42. 10.1186/1480-9222-16-11 PMC4177375 25276098

[24] Mercer TR, Mattick JS (2013). Structure and function of long noncoding RNAs in epigenetic regulation. Nat Struct Mol Biol.

[25] Morlando M, Ballarino M, Fatica A, Bozzoni I (2014). The role of long noncoding RNAs in the epigenetic control of gene expression. Chemmedchem.

[26] Pandey RR, Kanduri C (2011). Transcriptional and posttranscriptional programming by long noncoding RNAs. Progress in Molecular & Subcellular Biology.

[27] Mathieu EL, Belhocine M, Dao LT, Puthier D, Spicuglia S (2014). Functions of lncRNA in development and diseases. Med Sci.

[28] Desando G, Cavallo C, Sartoni F, Martini L, Parrilli A, Veronesi F, Fini M, Giardino R, Facchini A, Grigolo B (2013). Intra-articular delivery of adipose derived stromal cells attenuates osteoarthritis progression in an experimental rabbit model. Arthritis Research & Therapy.

[29] Gong L, Wang C, Li Y, Sun Q, Li G, Wang D (2014). Effects of human adipose-derived stem cells on the viability of rabbit random pattern flaps. Cytotherapy.

[30] Wang W, He N, Feng C, Liu V, Zhang L, Wang F, He J, Zhu T, Wang S, Qiao W (2015). Human adipose-derived mesenchymal progenitor cells engraft into rabbit articular cartilage. Int J Mol Sci.

[31] Ye X, Zhang P, Xue S, Xu Y, Tan J, Liu G (2014). Adipose-derived stem cells alleviate osteoporosis by enchancing osteogenesis and inhibiting adipogenesis in a rabbit model. Cytotherapy.

[32] Yu L, Zhang R, Li P, Zheng D, Zhou J, Wang J, Zhang B, Zhu C (2015). Erratum to: traditional Chinese medicine: Salvia miltiorrhiza enhances survival rate of autologous adipose tissue transplantation in rabbit model. Aesthet Plast Surg.

[33] Koufariotis LT, Chen YPP, Chamberlain A, Jagt CV, Hayes BJ (2015). A catalogue of novel bovine long noncoding RNA across 18 tissues. PLoS One.

[34] Wang Y, Xue S, Liu X, Liu H, Hu T, Qiu X, Zhang J, Lei M (2016). Analyses of long non-coding RNA and mRNA profiling using RNA sequencing during the pre-implantation phases in pig endometrium. Sci Rep.

[35] Arriagacanon C, Fonsecaguzmán Y, Valdesquezada C, Arzatemejía R, Guerrero G, Recillastarga F (2014). A long non-coding RNA promotes full activation of adult gene expression in the chicken α-globin domain. Epigenetics.

[36] Milisits G, Lévai A, Andrássybaka G, Romvári R. In vivo examination of fat deposition in growing rabbits selected for high and low body fat content. Agric Conspec Sci. 2003;68.

[37] Kim D, Langmead B, Salzberg SL (2015). HISAT: a fast spliced aligner with low memory requirements. Nat Methods.

[38] Franceschini A, Szklarczyk D, Frankild S, Kuhn M, Simonovic M, Roth A, Lin J, Minguez P, Bork P, Von MC (2013). STRING v9.1: protein-protein interaction networks, with increased coverage and integration. Nucleic Acids Res.

[39] Pertea M, Pertea GM, Antonescu CM, Chang TC, Mendell JT, Salzberg SL (2015). StringTie enables improved reconstruction of a transcriptome from RNA-seq reads. Nat Biotechnol.

[40] Pertea G (2010). Transcript assembly and quantification by RNA-Seq reveals unannotated transcripts and isoform switching during cell differentiation. Nat Biotechnol.

[41] Kong L, Zhang Y, Ye ZQ, Liu XQ, Zhao SQ, Wei L, Gao G (2007). CPC: assess the protein-coding potential of transcripts using sequence features and support vector machine. Nucleic Acids Res.

[42] Sun L, Luo H, Bu D, Zhao G, Yu K, Zhang C, Liu Y, Chen R, Zhao Y (2013). Utilizing sequence intrinsic composition to classify protein-coding and long non-coding transcripts. Nucleic Acids Res.

[43] Wang Liguo, Park Hyun Jung, Dasari Surendra, Wang Shengqin, Kocher Jean-Pierre, Li Wei (2013). CPAT: Coding-Potential Assessment Tool using an alignment-free logistic regression model. Nucleic Acids Research.

[44] Finn RD, Bateman A, Clements J, Coggill P, Eberhardt RY, Eddy SR, Heger A, Hetherington K, Holm L, Mistry J (2014). Pfam: the protein families database. Nucleic Acids Res.

[45] Li J, Ma W, Zeng P, Wang J, Geng B, Yang J, Cui Q (2015). LncTar: a tool for predicting the RNA targets of long noncoding RNAs. Brief Bioinform.

[46] Young MD, Wakefield MJ, Smyth GK, Oshlack A (2010). Gene ontology analysis for RNA-seq: accounting for selection bias. Genome Biol.

[47] Livak KJ, Schmittgen TD (2001). Analysis of relative gene expression data using real-time quantitative PCR and the 2(−Delta Delta C(T)) method. Methods.

[48] Huang DW, Sherman BT, Lempicki RA (2009). Systematic and integrative analysis of large gene lists using DAVID bioinformatics resources. Nat Protoc.

[49] Jayakodi M, Jung JW, Park D, Ahn YJ, Lee SC, Shin SY, Shin C, Yang TJ, Kwon HW (2015). Genome-wide characterization of long intergenic non-coding RNAs (lincRNAs) provides new insight into viral diseases in honey bees Apis cerana and Apis mellifera. BMC Genomics.

[50] Sun L, Goff LA, Trapnell C, Alexander R, Lo KA, Hacisuleyman E, Sauvageau M, Tazonvega B, Kelley DR, Hendrickson DG (2013). Long noncoding RNAs regulate adipogenesis. Pnas.

[51] Alvarezdominguez JR, Bai Z, Xu D, Yuan B, Lo KA, Yoon MJ, Lim YC, Knoll M, Slavov N, Chen S (2015). De novo reconstruction of adipose tissue transcriptomes reveals novel long non-coding RNAs that regulate Brown adipocyte development. Cell Metab.

[52] Wapinski O, Chang HY (2011). Long noncoding RNAs and human disease. Trends Cell Biol.

[53] Muret K, Klopp C, Wucher V, Esquerré D, Legeai F, Lecerf F, Désert C, Boutin M, Jehl F, Acloque H. Long noncoding RNA repertoire in chicken liver and adipose tissue. Genetics Selection Evolution Gse. 2017;49. 10.1186/s12711-016-0275-0 PMC5225574 28073357

[54] Liu Y, Sun Y, Li Y, Hao B, Xue F, Xu S, Hong X, Lei S, Ning Y, Chen J (2017). Analyses of long non-coding RNA and mRNA profiling using RNA sequencing in chicken testis with extreme sperm motility. Sci Rep.

[55] Ran M, Chen B, Li Z, Wu M, Liu X, He C, Zhang S, Li Z (2001). Systematic identification of long non-coding RNAs in immature and mature porcine testes. Biol Reprod.

[56] Xiao W, Hu Y, Tong Y, Cai M, He H, Liu B, Shi Y, Wang J, Qin Y, Lai S. Landscape of long non-coding RNAs in Trichophyton mentagrophytes-induced rabbit dermatophytosis lesional skin and normal skin. Functional & Integrative Genomics. 2018:1–10. 10.1007/s10142-018-0601-4 29560532

[57] Pauli A, Valen E, Lin MF, Garber M, Vastenhouw NL, Levin JZ, Fan L, Sandelin A, Rinn JL, Regev A (2012). Systematic identification of long noncoding RNAs expressed during zebrafish embryogenesis. Genome Res.

[58] Sigova AA, Mullen AC, Molinie B, Gupta S, Orlando DA, Guenther MG, Almada AE, Lin C, Sharp PA, Giallourakis CC (2013). Divergent transcription of long noncoding RNA/mRNA gene pairs in embryonic stem cells. Proc Natl Acad Sci U S A.

[59] Bao J, Wu J, Schuster AS, Hennig GW, Yan W (2013). Expression profiling reveals developmentally regulated lncRNA repertoire in the mouse male Germline1. Biol Reprod.

[60] Ren H, Wang G, Chen L, Jiang J, Liu L, Li N, Zhao J, Sun X, Zhou P (2016). Genome-wide analysis of long non-coding RNAs at early stage of skin pigmentation in goats (Capra hircus). BMC Genomics.

[61] Yu L, Tai L, Zhang L, Chu Y, Li Y, Zhou L (2017). Comparative analyses of long non-coding RNA in lean and obese pig. Oncotarget.

[62] Bravo-Sagua R, Mattar P, Díaz X, Lavandero S, Cifuentes M. Calcium sensing receptor as a novel mediator of adipose tissue dysfunction: mechanisms and potential clinical implications. Front Physiol. 2016;7. 10.3389/fphys.2016.00395 PMC5014866 27660614

[63] Wei N, Wang Y, Xu RX, Wang GQ, Xiong Y, Yu TY, Yang GS, Pang WJ (2015). PU.1 antisense lncRNA against its mRNA translation promotes adipogenesis in porcine preadipocytes. Anim Genet.

[64] Chen J, Liu Y, Lu S, Yin L, Zong C, Cui S, Qin D, Yang Y, Guan Q, Li X, Wang X (2016). The role and possible mechanism of lncRNA U90926 in modulating 3T3-L1 preadipocyte differentiation. International Journal of Obesity.

[65] Cooper DR, Carter G, Li P, Patel R, Watson JE, Patel NA (2014). Long non-coding RNA NEAT1 associates with SRp40 to temporally regulate PPARγ2 splicing during Adipogenesis in 3T3-L1 cells. Genes.

[66] Xiao T, Liu L, Li H, Yu S, Luo H, Li T, Wang S, Dalton S, Zhao RC, Chen R (2015). Long noncoding RNA ADINR regulates Adipogenesis by transcriptionally activating C/EBPα. Stem Cell Reports.

[67] Pang WJ, Lin LG, Xiong Y, Wei N, Wang Y, Shen QW, Yang GS (2013). Knockdown of PU.1 AS lncRNA inhibits adipogenesis through enhancing PU.1 mRNA translation. J Cell Biochem.

[68] Nie M, Deng ZL, Liu J, Wang DZ (2015). Noncoding RNAs, Emerging Regulators of Skeletal Muscle Development and Diseases. Biomed Research International.

[69] Wei S, Min D, Jiang Z, Hausman GJ, Zhang L, Dodson MV (2016). Long noncoding RNAs in regulating adipogenesis: new RNAs shed lights on obesity. Cellular & Molecular Life Sciences.

[70] Medeiros NI, Mattos RT, Menezes CA, Rcg F, Talvani A, Dutra WO, Riossantos F, Correaoliveira R, Jas G. IL-10 and TGF-β unbalanced levels in neutrophils contribute to increase inflammatory cytokine expression in childhood obesity. Eur J Nutr. 2017:1–10. 10.1007/s00394-017-1515-y 28735358

[71] Rodrigues Kathryna Fontana, Pietrani Nathalia Teixeira, Bosco Adriana Aparecida, Campos Fernanda Magalhães Freire, Sandrim Valéria Cristina, Gomes Karina Braga (2017). IL-6, TNF-α, and IL-10 levels/polymorphisms and their association with type 2 diabetes mellitus and obesity in Brazilian individuals. Archives of Endocrinology and Metabolism.

[72] Liu Y, Xu D, Yin C, Wang S, Wang M, Xiao Y (2018). IL-10/STAT3 is reduced in childhood obesity with hypertriglyceridemia and is related to triglyceride level in diet-induced obese rats. BMC Endocr Disord.

